# Effects of Chronic Alcohol Intake on the Composition of the Ensemble of Drug-Metabolizing Enzymes and Transporters in the Human Liver

**DOI:** 10.3390/jox15010020

**Published:** 2025-01-31

**Authors:** Kari A. Gaither, Guihua Yue, Dilip Kumar Singh, Julia Trudeau, Kannapiran Ponraj, Nadezhda Y. Davydova, Philip Lazarus, Dmitri R. Davydov, Bhagwat Prasad

**Affiliations:** 1 Department of Pharmaceutical Sciences, Washington State University, Spokane, WA 99202, USA; kari.gaither@wsu.edu (K.A.G.); guihua.yue@wsu.edu (G.Y.); dilip.singh@wsu.edu (D.K.S.);; 2 Department of Chemistry, Washington State University, Pullman, WA 99164, USA; kannapiran.ponraj@wsu.edu (K.P.);

**Keywords:** drug metabolism, human liver microsomes, cytochromes P450, alcohol consumption, tobacco smoking, proteomics, alcohol–drug interactions

## Abstract

In this study, to better understand the mechanisms of the profound impact of alcohol consumption on drug pharmacokinetics, efficacy, and toxicity, we characterized the alcohol-induced changes in the ensemble of drug-metabolizing enzymes and transporters (DMETs) in the human liver by performing global proteomic analysis of human liver microsomes from 94 donors. DMET protein levels were analyzed concerning alcohol consumption, smoking history, and sex using non-parametric tests, which were further strengthened by correlational analysis. To this end, we used a provisional index of alcohol exposure formulated based on the relative abundances of four marker proteins best correlating with the level of alcohol consumption. Alcohol-induced changes in the cytochrome P450 pool include significant increases in CYP2E1, CYP2B6, CYP2J2, and NADPH-cytochrome P450 reductase levels and the lowering of CYP1A2, CYP2C8, CYP2C9, CYP4A11, and cytochrome b_5_. Changes in UDP-glucuronosyltransferase (UGT) abundances comprise elevated UGT1A6, UGT1A9, and UGT2A1, and reduced UGT1A3, UGT1A4, UGT2B7, UGT2B10, and UGT2B15 levels. Tobacco smokers showed elevated CYP1A2, UGT1A6, and UGT2B4 and reduced FMO3, FMO4, and FMO5 levels, while in females, CYP1A2, UGT2B17, and UGT2B15 levels were lower, and UGT2A3 and STS were higher compared to males. The alcohol-induced changes in the DMET ensemble at the protein level reported herein provide deep insights into how alcohol impacts drug and xenobiotic metabolism.

## 1. Introduction

Alcohol and tobacco are widely used worldwide. In 2021, 60 million Americans reported excessive alcohol consumption, while 11.3 million smoked at least a pack of cigarettes per day [1]. Together, these substances ranked among the top ten risk factors for global disease burden, contributing to over 10 million combined deaths in 2019 [2,3]. A significant portion of alcohol-related fatalities are associated with drug–alcohol interactions. There are numerous known examples of changes in drug pharmacokinetics and pharmacodynamics due to both chronic and acute alcohol exposure, which can dramatically affect optimal dosing [4,5,6,7,8,9]. Therefore, consideration and prediction of alcohol–drug interactions (ADIs) are crucial for practical pharmacotherapy. While life-threatening ADIs with acute alcohol consumption are often highlighted in fatal co-intoxications with substances such as benzodiazepines and opiates [10,11,12], many drug interactions related to chronic alcohol exposure may remain unidentified.

The mechanisms governing the effects of alcohol on drug metabolism are not fully understood. Significant increases in cytochrome P450 2E1 (CYP2E1) observed in both alcoholics and moderate alcohol consumers represent one of the most notable effects of alcohol on protein expression [13,14]. However, the role of CYP2E1 in ADIs is generally considered insignificant due to its minor contribution to drug metabolism, except acetaminophen [4,15,16]. Nevertheless, the impact of alcohol-induced CYP2E1 on drug metabolism and other functions of cytochrome P450 enzymes (P450s) appears to be underestimated. For instance, CYP2E1 interactions with other P450s likely explain the alcohol-induced increase in the metabolism of CYP3A substrates such as diazepam and doxycycline [17,18], as well as phenytoin, tolbutamide, and warfarin [19,20], primarily metabolized by CYP2C9. Our studies provide evidence of a direct cause-and-effect relationship between the alcohol-dependent induction of CYP2E1 and its effects on CYP3A4, CYP1A2, and CYP2C19 activities [21,22].

Tobacco smoke contains over 7000 chemicals [23,24], which enter the lungs and subsequently make their way to the liver, where they are metabolized and can induce drug metabolizing enzyme (DME) expression [25]. Consequently, various drug interactions may occur in tobacco users [26]. Additionally, sex can also contribute to differences in drug-metabolizing and transporter (DMET) protein levels, with recent findings that UDP-glucuronosyltransferase (UGT) 2B17 levels are 2.6 times greater in males than in females [27].

In this study, the impact of alcohol and tobacco use is investigated, as well as sex, on DMET protein abundance in the human liver. Using high-throughput quantitative proteomics, we quantified DMET proteins in human liver microsomes (HLMs) from 94 donors with documented alcohol consumption and tobacco smoking histories. Large-scale studies of the DMET proteome in postmortem liver samples are scarce, typically limited in sample size, and often lack information on the effects of sex and lifestyle factors [28,29]. With our findings, we aim to contribute to a clinically translational understanding of the changes in the levels of proteins involved in drug metabolism and distribution, ultimately facilitating optimal dosing for precision medicine.

## 2. Materials and Methods

### 2.1. Chemicals and Reagents

Liquid chromatography−mass spectrometry (LC−MS)-grade acetonitrile, methanol, chloroform, and formic acid were procured from Fisher Scientific (Fair Lawn, NJ, USA), while acetone was obtained from Sigma-Aldrich (St. Louis, MO, USA). Ammonium bicarbonate (98% pure), dithiothreitol, iodoacetamide, and MS-grade trypsin were purchased from Thermo Fisher Scientific (Rockford, IL, USA). The bicinchoninic acid (BCA) kit was obtained from Pierce Biotechnology (Rockford, IL, USA).

### 2.2. Human Liver Microsomes

The majority of the liver samples used in this study (*n* = 88) were sourced from biobanks established in the Prasad and Lazarus Laboratories. Additionally, six liver tissue samples from moderate-to-heavy alcohol consumers were procured from BioIVT Corporation (Westbury, NY, USA). The primary inclusion criterion was a documented history of alcohol intake. One aim of selection was to identify and include donors with a history of heavy alcohol consumption while excluding those with illicit substance use as potential confounders where possible. The demographic characteristics of our study population are summarized in Table 1.

Microsomes from 71 liver specimens from the Lazarus Laboratory were prepared via differential centrifugation, as described previously [30]. Preparation of microsomal fractions from 17 liver specimens from Prasad Laboratory was performed, as described by Nelson et al. with minor modifications [31]. Frozen liver tissue specimens (0.5–1 g) were covered with 0.1 M potassium phosphate buffer, pH of 7.4, containing 0.125 M KCl, 0.25 M sucrose, and 1.0 mM EDTA (Buffer A) at room temperature and allowed to thaw. After decanting the buffer, the tissue was minced with scissors on ice and supplemented with 2.5 volumes of ice-cold Buffer A containing 0.25 mM phenylmethylsulfonyl fluoride (PMSF). The mixture was homogenized on ice with ten strokes in a glass homogenizer using a motorized Teflon pestle. After diluting the homogenate to 7–8 volumes of the sample weight with ice-cold PMSF-containing Buffer A, it was centrifuged at 10,500× *g* for 40 min. The pellet was discarded, and the supernatant was centrifuged at 118,000× *g* for 90 min. The upper lipid layer and the supernatant were discarded. The pellet was resuspended in 0.1 M Na-HEPES buffer containing 60 mM KCl and 0.25 M sucrose, pH of 7.4 (Buffer B), to reach 1.5–2 volumes of the sample weight and centrifuged at 118,000× *g* for 90 min. The pellet was resuspended in Buffer B (1 mL per 1 g of tissue) using a syringe and plastic pestle in a 1.5 mL Eppendorf tube and stored at −80 °C. 

### 2.3. Trypsin Digestion and Sample Preparation for Proteomics Analysis

HLMs were analyzed for protein content using a BCA kit according to standard vendor protocols and then digested following an optimized trypsin digestion protocol described previously [32]. Briefly, 1 mg/mL protein samples (80 µg) in 100 mM ammonium bicarbonate, pH of 7.8, were reduced and denatured by adding 250 mM dithiothreitol and incubating at 95 °C for 10 min with gentle shaking. After bringing samples to room temperature, proteins were alkylated by adding 100 mM iodoacetamide (incubated in the dark for 30 min). Protein precipitation was initiated by adding ice-cold acetone at −80 °C for 1 h. Samples were then centrifuged at 16,000× *g* for 10 min at 4 °C and the supernatant was discarded. Samples were washed with ice-cold methanol followed by another round of high-speed centrifugation at 16,000× *g* for 10 min at 4 °C. The supernatant was removed, and the pellet was dried for 30 min at room temperature. The pellet was then resuspended in 60 μL ammonium bicarbonate buffer (50 mM, pH of 7.8). Protein digestion occurred by adding 20 μL trypsin (50:1, protein/trypsin ratio) with gentle shaking at 37 °C for 16 h and was quenched with 5 µL of 5% formic acid in water. The sample was then centrifuged at 16,000× *g* for 10 min at 4 °C and stored at −80 °C until LC-MS analysis. 

### 2.4. LC-MS Data Acquisition

Global quantitative proteomics analysis was performed using the Easy Spray 1200 series nanoLC coupled with an Orbitrap Q-Exactive Mass Spectrometer (Thermo Fisher Scientific, Waltham, MA, USA). One µL of protein digest sample (1 µg/µL) was injected and peptide separation was achieved using a Thermo Scientific Acclaim Pepmap RSLC C18 25 cm × 75 µm (2 µm, 100 Å) column with a mobile phase consisting of 0.1% formic acid in water (A) and 80% acetonitrile with 0.1% formic acid (B). The flow rate was set to 300 nL/min with a 35 min gradient as follows: 0–2 min (0–10% B), 2–27 min (10–45% B), 27–28 min (45–100% B), and 28–35 min (100% B).

The eluted peptides were detected in data-independent acquisition (DIA) mode. The spray voltage was set to 1.7 kV with 300 °C capillary heat. The MS1 scan range was set to *m*/*z* 348–1100 with a mass resolution of 60,000, an auto gain control (AGC) target of 3 × 10^6^, and a maximum injection time of 55 msec. MS2 was set to a resolution of 30,000, AGC of 1 × 10^6^, normalized collision energy of 30, and a maximum injection time of 55 msec. The DIA had a variable isolation window set to 25 *m*/*z* spanning the 350–400 mass range, 20 *m*/*z* spanning the 400–870 mass range, and 40 *m*/*z* spanning the 870–1110 mass range.

### 2.5. Proteomics Data Analysis

DIA-NN (version 18.1.1) (https://github.com/vdemichev/DiaNN accessed on 23 January 2025) was used for library-free analysis [33]. Deep learning-based in silico spectral library generation was enabled with the human FASTA database. The identified peptides had a maximum number of missed cleavages set to 1 as the default. Fixed modification included carbamidomethylation and N-terminal methionine excision, while variable modification included the oxidation of methionine residues and acetylation of protein N termini. Peptides were identified with a 1% false discovery rate. An unrelated run was selected, and all other parameters used the default settings.

To account for batch-to-batch or interlaboratory technical variability during the preparation of subcellular fractions, we normalized DMET proteins to the sum of a set of 75 proteins with known localization in the microsomal membrane or lumen (Supplementary Table S1). For this normalization, the raw MS intensities (MSI) for each (*i*-th) protein in each (*j*-th) HLM sample were first normalized to the total intensities of all 75 proteins in that sample, as follows:

(1) $$MSI_{i,j}^{norm}=\frac{MSI_{i,j}}{\sum_{k=1}^{75}MSI_{k,j}}$$

We employed the total protein approach (TPA) to compare protein abundances between different treatment groups quantitatively [34]. This method yields accuracy similar to that obtained by targeted proteomics for protein quantification while allowing for a broader coverage of proteins detected [32]. Using this approach makes it possible to determine the protein concentration (mg individual protein per mg total protein) of individual proteins within a sample, as follows:

$${\left[\mathrm{Protein}\right]}_{i}=\frac{MSI_{i}^{norm}}{MSI_{total}^{norm}\times MW_{i}}$$

where $MSI_{i}^{norm}$ is the normalized MS1 intensity defined as the normalized (see Equation (1)) sum of the MS1 spectral intensities of all peptides identified to match the sequence of i-th protein; $MSI_{total}^{norm}$ is the sum of all normalized MS spectral intensities for all proteins in a particular sample; and MW_i_ is the molecular weight of the i-th protein. Pathways were determined using the STRING database (www.string-db.org accessed on 23 January 2025) with *homo sapiens* background [35].

### 2.6. Non-Parametric Statistical Analysis and Data Visualization

To assess alcohol-induced differences in DMET protein abundance, individual-derived HLMs were classified into the following five groups based on alcohol intake history: (1) non-drinking control group, including those with a past history of drinking, (2) light alcohol drinkers, (3) social drinkers, (4) moderate alcohol drinkers, and (5) heavy alcohol drinkers. Light alcohol consumption was defined as one alcoholic drink or less per day, moderate as two to three drinks per day, and heavy as more than three drinks per day. The social alcohol category lacks a definitive quantity consumed and is ordered between the light and moderate groups. Statistical analysis was performed using GraphPad Prism 8.4.3 (GraphPad Software, La Jolla, CA, USA) which generated Volcano plots and bar charts. Significant differences in DMET proteins were assessed using the Student’s *t*-test with Welch’s correction for unequal variance, with a *p*-value of <0.05 considered significant. BioRender (Toronto, ON, Canada) was used to produce figures and InteractiVenn [36] was used to create VENN diagrams. Pie charts were produced in Microsoft Excel for Microsoft 365 MSO (Version 2402 Build 16.0.17328.20282) 64-bit (Microsoft, Redmond, WA, USA). Correlation analysis was performed using SpectraLab data analysis software version 3.1.1 (http://cyp3a4.chem.wsu.edu/spectralab.html accessed on 23 January 2025).

### 2.7. Analysis of Correlations of Protein Abundances with the Level of Alcohol Consumption

In our further analysis of the effects of alcohol exposure on the HLM proteome, we established an Alcohol Consumption Grade (ACG) scale to numerically assess the level of alcohol exposure among liver donors. Donors categorized as “non-drinkers”, “former drinkers”, “light drinkers”, “social drinkers”, “low-to-moderate drinkers”, “moderate drinkers”, and “heavy drinkers” were assigned grades of 0, 0.5, 1, 1.5, 2, 3, and 4, respectively. These assignments were based on available demographic records for the donors. This gradation slightly differs from the one used in the initial non-parametric analysis of the proteomics data; specifically, we grouped former drinkers separately to avoid exaggerating their claims of abstinence and divided moderate drinkers into two groups, “low-to-moderate drinkers” consuming two drinks per day and “moderate drinkers” consuming three drinks per day.

We then focused on a subset of 75 DMETs and endoplasmic reticulum (ER)-stress-related proteins with known localization in the microsomal membrane or lumen (Supplementary Table S1). This subset included P450s and their interaction partners (cytochrome b5, CPR, PGRMC1, and heme oxygenases), other microsomal drug-metabolizing enzymes (UGTs, FMOs, glutathione S-transferases, and esterases such as CES2 and CES3), as well as proteins involved in the cellular response to ER stress—chaperones HSPA5, HSPA9, and HSPA90B1, protein disulfide isomerases, ER oxidoreductases ERO1A and ERO1B, transitional ER ATPase (VCP), and some other relevant proteins (PGRMC2, STS, AADAC, ABCB6). To evaluate the correlation between the ACG scores and variations in protein abundances, we calculated vectors of relative protein abundance (VRA) for each of the 75 proteins. For these calculations, we used the MS intensities normalized to the total intensities of all 75 selected proteins in each sample (see Equation (1) above). After normalization, the averaged normalized intensity for each protein across all 94 HLM samples was assessed and used to calculate the VRA value for each (*i*-th) protein in each (*j*-th) HLM sample, as follows:

$$VRA_{i,j}=\frac{94\times MSI_{i,j}^{norm}}{\sum_{k=1}^{94}MSI_{i,k}^{norm}}$$

The resulting 75 VRA vectors, reflecting relative differences in protein abundances between HLM samples, were used to determine their linear combination approximating the vector of Alcohol Consumption Grade. In this analysis, we approximated the ACG vector as a linear combination of several VRAs using the multidimensional linear least-squares regression algorithm, as described earlier [37]. This algorithm was applied sequentially to every possible combination of 2–4 proteins to better approximate the ACG vector and to find a numerical scale best reflecting the apparent alcohol exposure of the liver donors (Provisional Index of Alcohol Exposure, PIAE). The search algorithm described above was implemented using our SpectraLab data analysis software. The found PIAE vector was then used to probe its correlations with VRA for all 75 selected proteins.

## 3. Results

### 3.1. Effects of Alcohol Consumption and Tobacco Smoke on Global Proteome

**Figure 1:**
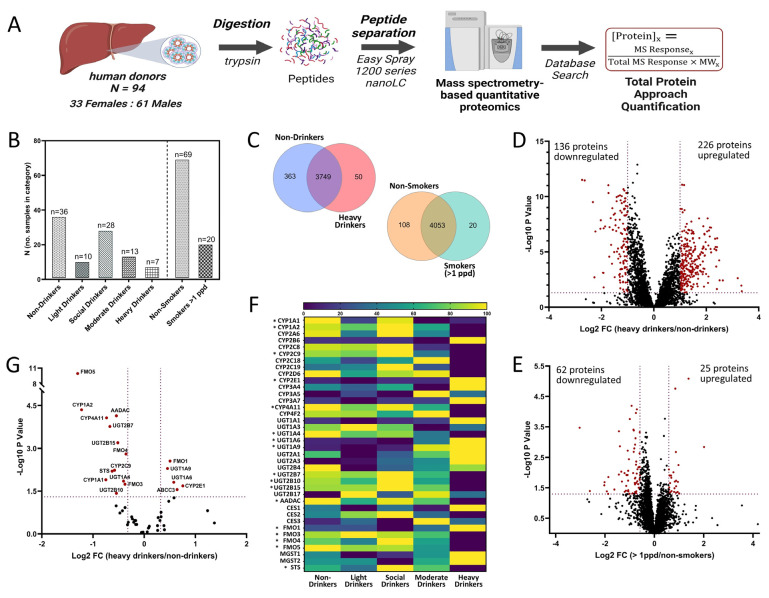
Global proteomics analysis of human liver microsomes prepared from 94 individuals with known alcohol intake histories. (**A**) Mass spectrometry-based workflow scheme of human liver microsomes (N = 94, 33 females and 61 males); (**B**) number of HLM specimens from individuals in each alcohol and smoking history category; (**C**) Venn diagrams of proteins detected and overlapping in non-drinkers vs. heavy drinkers and non-smokers vs. individuals who smoked > 1 ppd; (**D**) differentially expressed proteins in HLMs derived from 7 heavy drinking individuals and 31 non-drinking individuals (*p*-value < 0.05, fold change cutoff of 2.0); (**E**) differentially expressed proteins in HLMs from 20 individuals who smoked > 1 ppd and 69 non-smokers (*p*-value < 0.05, fold change cutoff of 1.5); (**F**) heatmap of CYP, UGT, and non-P450, non-UGT proteins resulting from label-free quantitative proteomic analysis of individual-derived human liver microsomes, based on the normalized mean protein levels for each DMET across the alcohol use categories. Proteins with significantly different levels of expression in HLMs from the heavy drinkers compared to the non-drinkers are indicated with an asterisk (*p*-value < 0.05); (**G**) differential expression of DMET proteins in individual-derived HLMs from heavy drinkers as compared to non-drinkers. (*p*-value < 0.05, fold-change cutoff of 1.25).

The HLMs from 94 individuals (demographics summarized in Table 1) were stratified by alcohol intake and analyzed using LC-MS, as outlined in Figure 1A. A total of 4198 proteins were detected across all groups. Comparing the global proteomics data for the non-drinking control group and the heavy drinking group revealed 4162 proteins with the majority overlapping; over 360 proteins were unique to HLMs from non-drinkers, while 50 were unique to HLMs from heavy drinkers (Figure 1C). In contrast, when the non-smoking group was compared to the >1 ppd group, only 128 proteins out of the 4181 detected were unique to the two groups; 108 proteins in the non-smoking control group and 20 in the smoking group (Figure 1C). As illustrated in Figure 1D, 226 proteins were significantly upregulated and 136 significantly downregulated (FC ≥ 2) in HLMs from the heavy drinkers as compared to the non-drinkers (Figure 1D). Overall, just 25 proteins showed significantly elevated levels in the HLMs of moderate to heavy smokers (>1 ppd) compared to non-smoking controls, while 62 proteins were significantly downregulated (FC ≥ 1.5) (Figure 1D).

Notably, the number of significant changes in protein levels decreased with lower levels of alcohol consumption. When the protein expression in HLMs from moderate drinkers was compared to non-drinkers, the number of significantly upregulated proteins was approximately 50% lower, with fewer than 15 significantly downregulated, while less than 50 proteins exhibited significantly altered levels in either the social or light drinker groups compared to the non-drinking control group (Supplementary Figure S1A). The STRING analysis of the significantly upregulated proteins from our dataset revealed pathways involved in protein degradation, amino acid maintenance, and energy regulation that were upregulated with heavy alcohol consumption, while the downregulated pathways included those related to liver health, bile acid homeostasis, metabolism of complex carbohydrates, drug metabolism by P450s, cholesterol homeostasis, and oxidative phosphorylation (Supplementary Figure S2).

### 3.2. Effects of Alcohol Intake on DMET Proteome Abundance and Composition

To investigate the impact of alcohol consumption on DMET protein expression, we assessed the absolute amounts of proteins using the TPA approach across each alcohol intake group compared to the non-drinking control group. A stark difference was evident when evaluating the number of DMET proteins with significantly altered levels in each alcohol intake category compared to the non-drinking control group (Figure 1F). While many DMET proteins showed significantly altered levels in HLMs from heavy drinkers compared to non-drinking controls, significant changes in the DMET protein levels were minimal in the HLMs of light, social, and moderate drinkers compared to non-drinkers (Figure 1F, Supplementary Figure S1B). Consequently, further analyses of the effects of alcohol on the liver proteome excluded the light, social, and moderate alcohol intake groups. Overall, most DMETs with significantly altered protein levels in HLMs from heavy alcohol drinkers exhibited decreased protein abundance compared to non-drinkers, with the exceptions of CYP2E1, UGT1A6, UGT1A9, FMO1, and MRP3, which were significantly elevated with heavy alcohol intake (FC > 1.25) (Figure 1G). The mean protein levels of P450s, UGTs, and non-P450, non-UGT drug metabolizing enzymes quantified in HLMs from the non-drinking control and heavy alcohol intake groups are reported with standard deviations and significance levels in Table 2.

**Figure 2:**
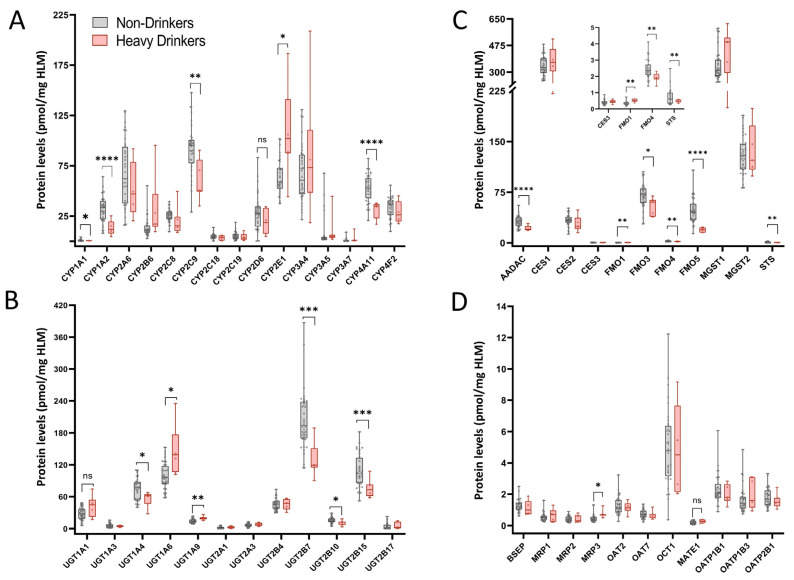
Differential DMET protein expression with heavy alcohol intake. (**A**) Protein levels of major drug metabolizing CYPs in HLMs from heavy drinkers compared to non-drinkers; (**B**) UGT protein levels in HLMs from heavy drinkers compared to non-drinkers; (**C**) protein levels of major non-P450, non-UGT enzymes in HLMs of individuals with heavy alcohol intake compared to non-drinkers; (**D**) transporter protein levels in HLMs of heavy drinkers compared to non-drinkers. Samples were analyzed for significance using the Student’s *t*-test with Welch’s correction for unequal variance. *p*-Value * < 0.05, ** < 0.01, *** < 0.001, **** < 0.0001. Absolute protein concentration for each DMET protein, expressed as pmol/mg of total HLM protein, was calculated via the TPA method.

Differential P450 expression was observed in HLMs of heavy drinkers compared to non-drinkers (Figure 2A, Table 2). Notably, protein levels of CYP1A1 (FC 0.59), CYP1A2 (FC 0.43), CYP2C9 (FC 0.67), and CYP4A11 (FC 0.60) were significantly reduced with heavy alcohol intake, whereas CYP2E1 protein levels increased significantly (FC 1.7). A decrease in CYP2D6 levels approached significance (FC 0.68, *p*-value = 0.10) in heavy drinkers.

**Figure 3:**
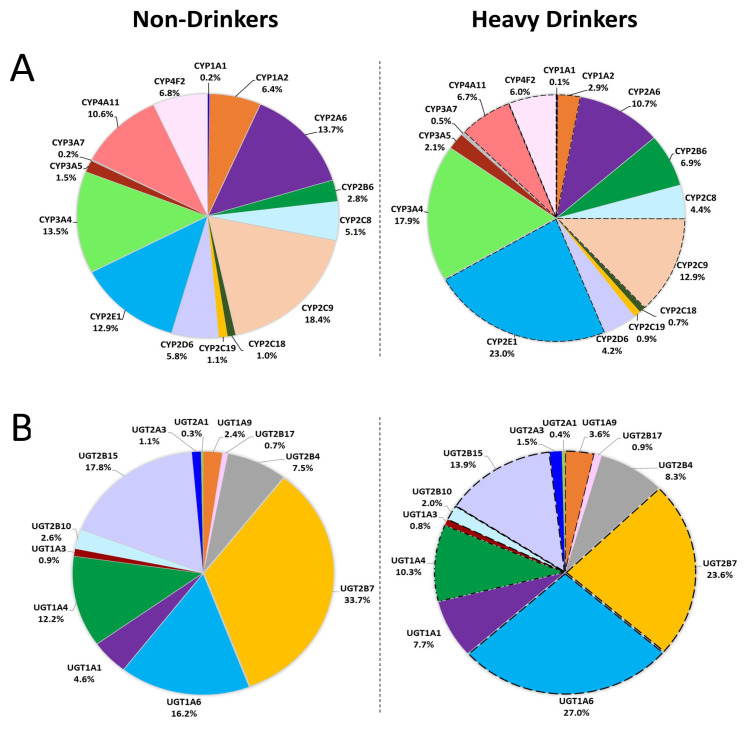
Shifts in the overall composition of proteins related to drug metabolism and disposition with excessive alcohol intake. (**A**) CYP protein composition in HLMs from heavy drinkers compared to non-drinkers; (**B**) UGT protein composition in HLMs of heavy drinkers compared to non-drinkers. Absolute protein levels were calculated using the TPA method, and significance was assessed using the Student’s *t*-test with Welch’s correction for unequal variance; dashed lines indicate a *p*-value < 0.05 for altered protein levels in HLMs of individuals with heavy alcohol intake compared to the non-drinking control group.

As shown in Figure 3A, these significant changes led to a considerable alteration in the composition of the P450 pool. The fraction of CYP2E1 increased from 12.9% in non-drinkers to 23% in heavy drinkers, while CYP1A1 and CYP1A2 decreased from 0.2% to 0.1% and 6.4% to 2.9%, respectively. Similarly, CYP2C9 and CYP2D6 decreased from 18.4% and 5.8% in non-drinkers to 12.9% and 4.2%, in heavy drinkers. CYP4A11 and CYP4F2 levels also decreased, from 10.6% and 6.8% in non-drinkers to 6.7% and 6.0%, respectively. CYP4F11, CYP4F3, and CYP8B1 underwent a slight but statistically significant reduction with heavy alcohol intake, shifting from 1.8 to 1.7%, 2.2 to 1.8%, and 5.6 to 4.6%, respectively.

Heavy alcohol consumption also significantly altered UGT levels (Figure 2B, Table 2). Protein levels of UGT1A4 (FC 0.75), UGT2B7 (FC 0.63), UGT2B10 (FC 0.68), and UGT2B15 (FC 0.7) were significantly reduced, while UGT1A6 (FC 1.5) and UGT1A9 (FC 1.4) were significantly elevated. A reduction in UGT1A1 approaching significance (FC 1.5, *p* = 0.06) was also observed.

The alcohol-induced changes in UGT abundances resulted in substantial alterations in the UGT composition (Figure 3B). In non-drinkers, UGT2B7 accounted for nearly 34% of all UGTs, followed by UGT2B15 (17.8%), UGT1A6 (16.2%), and UGT1A4 (12.2%). In heavy drinkers, the UGT1A6 fraction increased by 11% to 27% and the content of UGT2B7 decreased by ~10% to 23.6%, while UGT2B15 and UGT1A4 were reduced to 13.9% and 10.3%, respectively, and UGT1A9 increased from 2.4% in non-drinkers to 3.6% in heavy drinkers.

The expression pattern of the non-P450 and non-UGT enzymes was also altered by heavy alcohol intake (Figure 2C, Table 2). Protein levels of AADAC (FC 0.69), FMO3 (FC 0.77), FMO4 (FC 0.78), FMO5 (FC 0.41), and STS (FC 0.65) significantly decreased, while FMO1 (FC 1.4, *p* < 0.01) levels increased significantly.

Altered levels of transporter proteins were noted in the HLMs of heavy drinkers (Figure 2D), the MRP3 levels significantly increased (FC 1.6), and MATE1 levels increased, approaching significance (FC 1.4, *p* = 0.07).

Further analysis excluding specimens from individuals who smoked >1 ppd (Supplementary Table S2) showed consistent trends in altered DMET protein expression levels, except for CYP2C8, which significantly decreased (FC 0.6) in non-smoking heavy drinkers.

### 3.3. Establishing a Provisional Index of Alcohol Exposure and Its Use for In-Depth Analysis of the Alcohol Effects on HLM Proteome

Although the analysis of differences between the five categories of donors classified by alcohol consumption provided valuable results on the effects of alcohol on the HLM proteome, this approach suffers from approximation due to the voluntary and approximate reporting of the level of alcohol consumption by liver donors. To improve the gradation of the liver samples and increase the robustness of our analysis, we sought to establish a scale of alcohol consumption based on the abundance of the protein markers of alcohol exposure in HLMs.

To this end, we selected 75 proteins known to localize in the microsomal membrane or lumen (Supplementary Table S1) to calculate vectors of relative abundance (VRA) reflecting the differences in protein abundances among HLM samples. These vectors were then probed for their correlations with the vector of apparent alcohol consumption grade (ACG), where the grades from 0 to 4 were assigned according to the reported alcohol intake of the liver donors (see Materials and Methods).

We assessed the correlation between the ACG with the relative abundance vectors of each selected protein. The strongest correlation (R^2^ = 0.23) was found with FMO5, followed by HSPA5, a chaperone involved in the cellular response to the ER stress (R^2^ = 0.215), and VCP, a protein responsible for exporting misfolded proteins from the ER to the cytoplasm (R^2^ = 0.21). While the effects of alcohol on VCP and FMO5 have not been documented, the alcohol-induced increase in HSPA5 is well-established [38,39,40].

We then explored combinations of two–four proteins to better approximate the ACG vector. The best two-protein combination, HSPA5 and PDIA3, yielded an R^2^ of 0.37. The best three-protein combination (HSPA5, PDIA3, and CES2) had an R^2^ of 0.44, while the best four-protein combination (HSPA5, PDIA3, CES2, and P4HB) reached an R^2^ of 0.50. The coefficients for the VRAs of these proteins were 5.06, 2.35, −7.8, and 1.19, respectively.

The found combination of proteins contains three known markers of ER stress—endoplasmic reticulum chaperone HSPA5 and the two protein disulfide isomerases of P4HB and PDIA3. Notably, despite the widely recognized role of these proteins in the cellular response to ER stress, no correlation of their abundance with alcohol exposure has been previously demonstrated. Interestingly, the two disulfide isomerases in this combination show opposite signs—the contribution of P4HB is positive, whereas that of PDIA3 is negative. These opposite signs of contributions of two PDIAs may be needed to distinguish alcohol exposure from other possible ER stress inducers.

**Figure 4:**
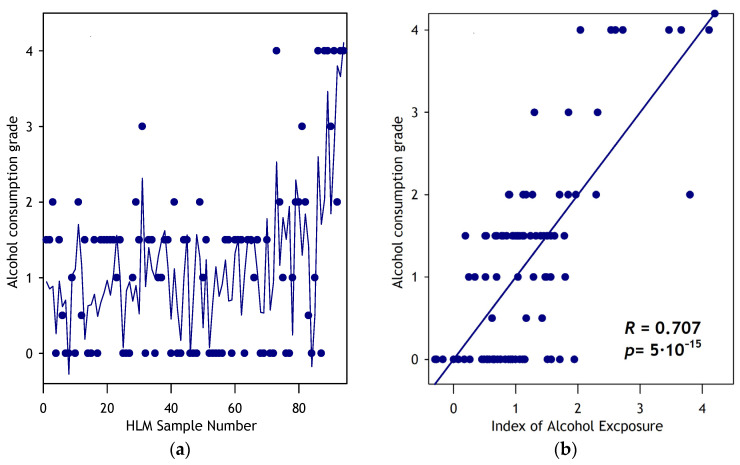
Approximation of the apparent alcohol exposure grade (ACG) with the provisional index of alcohol exposure (PIAE). The **left panel** (**a**) shows the plots of the grade of alcohol exposure (circles) and the index of alcohol exposure (solid line) for all 94 HLM samples sorted by increasing the relative abundance of HSPA5. The **right panel** (**b**) shows the same data as a plot of ACG versus PIAE.

The correlation of this combination of four VRAs, which we termed the Provisional Index of Alcohol Exposure (PIAE), with the ACG is illustrated in Figure 4. Figure 4a shows the correlation of PIAE with ACG across 94 HLM samples, ordered by the increasing abundance of HSPA5. Figure 4b depicts the plot of ACG versus PIAE. The correlation is pronounced, with a *p*-value of 3 × 10^−15^, indicating that PIAE effectively correlates the relative abundances of microsomal proteins with alcohol exposure levels in HLM donors. The results of this correlational analysis are shown in Table 3, which exemplifies the microsomal proteins exhibiting the *t*-test *p*-value below 0.05.

Although many of the correlations identified in the initial non-parametric analysis were confirmed by the PIAE strategy, several notable differences emerged between the results of the two approaches, as illustrated in Table 3. In addition to the upregulation of UGT1A6 and UGT1A9 observed in the non-parametric analysis, we detected a statistically significant upregulation of UGT2A1. The PIAE analysis also added UGT1A3 to the list of downregulated proteins while confirming the downregulation of UGT1A4, UGT2B7, and UGT2B15. Alongside the upregulation of CYP2E1 detected by both methods, the PIAE approach identified statistically significant upregulation of CYP2J2 and CYP2B6. Importantly, it also revealed a significant upregulation of NADPH-cytochrome P450 reductase (POR) consistent with the results of non-parametric analysis that detected a significant increase in POR levels (FC 1.5) with heavy alcohol consumption. Furthermore, the PIAE analysis confirmed the downregulation of CYP1A1, CYP1A2, CYP2C9, and CYP4A11. It also validated the significant downregulation of CYP7B1 (FC 0.68) and extended the list of downregulated proteins to include CYP2C8 and CYP4F11. Additionally, it indicated an alcohol-induced decrease in the abundance of cytochrome *b*_5_ (CYB5A), heme oxygenase 1 (HMOX1), and microsomal glutathione S-transferase MGST1. The PIAE analysis also confirms the results of non-parametric analysis indicating a significant decrease in MGST3 (FC 0.71) and a significant increase in GSTO1 (FC 1.97) with heavy alcohol consumption. Table 4 summarizes the alcohol-induced changes in the abundance of UGTs, cytochromes P450, and their redox partners identified by PIAE analysis.

### 3.4. Effects of Tobacco Smoke on DMET Abundance

**Figure 5:**
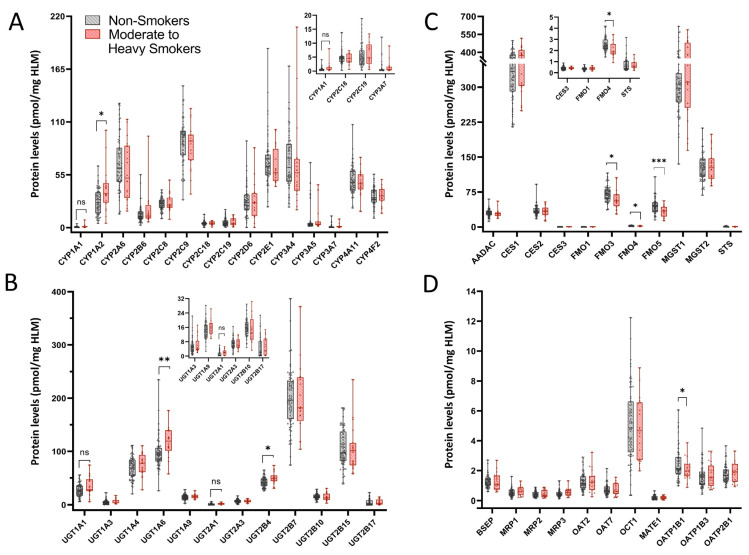
Differential DMET protein expression with moderate to heavy tobacco use. (**A**) Protein levels of major drug metabolizing CYPs in HLMs from >1 ppd smokers compared to non-smokers; (**B**) UGT protein levels in HLMs from >1 ppd smokers compared to non-smokers; (**C**) protein levels of major non-P450, non-UGT enzymes in HLMs of >1 ppd smokers compared to non-smokers; (**D**) transporter protein levels in HLMs of >1 ppd smokers compared to non-smokers. Samples were analyzed for significance using the Student’s *t*-test with Welch’s correction for unequal variance. *p*-Value * < 0.05, ** < 0.01, *** < 0.001. Absolute protein concentration for each DMET protein, expressed as pmol/mg of total HLM protein, was calculated via the TPA method.

Absolute protein concentrations for each DMET protein, expressed as pmol/mg of total HLM protein, were calculated using the TPA method to assess the impact of moderate-to-heavy smoking (>1 ppd) on DMET protein expression. In HLMs from >1 ppd tobacco smokers compared to non-smokers, the CYP1A2 protein levels were upregulated significantly (FC 1.45) and CYP1A1 protein levels were trending higher (FC 1.7, *p* = 0.095) with tobacco use (Figure 5A, Table 2). The expression of several UGTs was significantly increased, including UGT1A6 (FC 1.2) and UGT2B4 (FC 1.2) in HLMs from >1 ppd tobacco users compared to non-smokers, while UGT1A1 (FC 1.3, *p* = 0.08) and UGT2A1 (FC 1.6, *p* = 0.065) levels approached significance in tobacco users (Figure 5B, Table 2). Non-P450, non-UGT enzyme levels showed significant decreases in FMO3 (FC 0.82), FMO4 (FC 0.81), and FMO5 (FC 0.74) (Figure 5C, Table 2) with smoking. The transporter protein expression remained stable except for OATP1B1 (FC 0.83), which exhibited a significant decrease in protein levels with smoking (Figure 5D). When the analysis was performed to account for the potential confounding effects of alcohol by excluding heavy drinkers, similar trends in DMET protein levels were observed (Supplementary Table S2).

### 3.5. Effects of Sex on DMET Abundance

**Figure 6:**
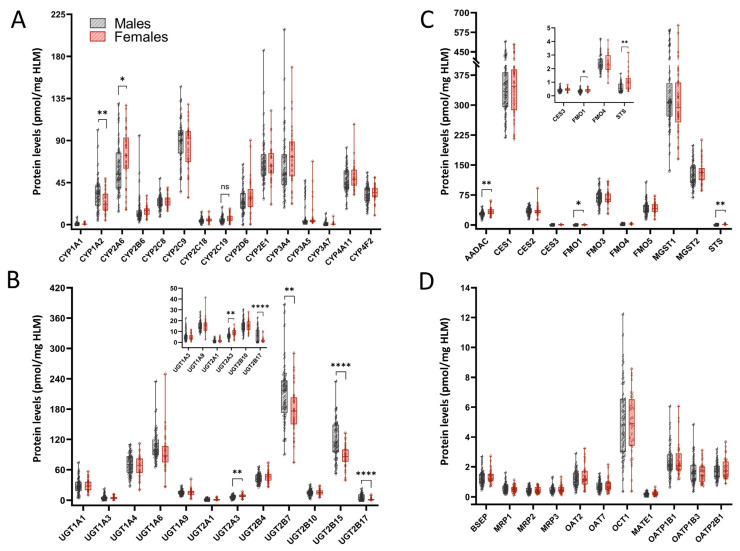
Differential DMET protein expression with sex. (**A**) Protein levels of major drug metabolizing CYPs in HLMs from female donors compared to males; (**B**) UGT protein levels in HLMs from female compared to male donors; (**C**) protein levels of major non-P450, non-UGT enzymes in HLMs from female donors compared to males; (**D**) transporter protein levels in HLMs from female donors compared to males. Samples were analyzed for significance using the Student’s *t*-test with Welch’s correction for unequal variance. *p*-Value * < 0.05, ** < 0.01, **** < 0.0001. Absolute protein concentration for each DMET protein, expressed as pmol/mg of total HLM protein, was calculated via the TPA method.

The comparison of P450 expression in HLMs from females and males revealed significantly lower CYP1A2 (FC 0.74) protein levels in females, while CYP2A6 (FC 1.2) levels were significantly higher, and CYP2C19 (FC 1.3, *p* = 0.054) levels were trending higher in females (Figure 6A, Table 2). Several UGTs, including UGT2B7 (FC 0.85, *p* < 0.01), UGT2B15 (FC 0.72), and UGT2B17 (FC 0.27), were significantly lower in HLMs from females compared to males (Figure 6B, Table 2). Conversely, UGT2A3 (FC 1.35) protein levels were significantly elevated in females. Analysis of non-P450, non-UGT enzyme levels in HLMs from females compared to males (Figure 6C, Table 2) revealed significantly elevated protein levels of AADAC (FC 1.2, *p* < 0.01), FMO1 (FC 1.15, *p* < 0.05), and STS (FC 1.7, *p* < 0.01) in females. The transporter protein levels in HLMs from females were comparable to those of males with no significant differences observed (Figure 6D, Table 2).

## 4. Discussion

Alcohol and tobacco use are prevalent; however, the knowledge of the effects of both on the abundance of DMET proteins remains limited. Accurately assessing potential ADI is critical for establishing a safe and effective drug profile across the population [41,42,43]. In this study, we employed TPA-based global proteomics to compare the abundance and composition of DMET proteins in HLM preparations from 94 donors with documented alcohol consumption and tobacco histories, exploring the effects of these factors on the human drug-metabolizing system. We detected over 4000 proteins in our HLM samples and analyzed DMET protein levels concerning alcohol consumption, smoking history, and sex using non-parametric tests (*p* ≤ 0.05; >1.25 FC). The examination of alcohol-induced changes was further enhanced by correlational analysis, using an alcohol consumption grade (ACG) scaling from 0 to 4 to establish a set of protein markers. We elaborated a PIAE scale based on the relative abundances of four proteins (HSPA5, PDIA3, P4HB, and CES2) that best correlated with ACG. This PIAE index was then utilized to find correlations with DMET protein abundances, allowing for corroborating and extending the conclusions from the initial non-parametric analysis.

Our results demonstrate significant alcohol-induced changes in the composition of the cytochrome P450 pool in HLMs. Consistent with the literature [7,13,14,16,44], we observed a significant upregulation of CYP2E1 with alcohol consumption, averaging a 1.7-fold increase in heavy drinkers. In addition to this well-documented effect, our correlational analysis revealed significant increases in the abundances of CYP2B6 and CYP2J2, mirroring the significant increase in POR levels, which has not been previously reported in the literature. The increase in POR levels may, in part, contribute to the overall enhancement of drug metabolism associated with alcohol consumption, as noted in several other studies [4,9,20,45]. Notably, we did not detect any alcohol-induced increase in CYP3A4 or CYP2A6 abundance, contrary to suggestions in the literature based on animal models and in vitro data [46,47,48].

Our novel proteomics analysis and PIAE index uncovered a significant decrease in the levels of CYP1A1, CYP1A2, CYP2C8, CYP2C9, CYP4A11, CYP4F11, and CYP7B1 proteins due to chronic alcohol exposure. The average levels of CYP1A2, CYP2C9, and CYP4A11 were reduced by 2.3-, 1.5-, and 1.7-fold, respectively, in heavy alcohol consumers, which could significantly impact the pharmacokinetics of various drugs metabolized by these enzymes. Furthermore, the alcohol-induced decrease in CYP4A11 abundance, which is involved in the synthesis of eicosanoids, may affect the 20-HETE signaling pathways and contribute to mechanisms of alcohol-induced hypertension. The observed decrease in cytochrome *b*_5_ levels with alcohol exposure may also have important physiological implications as interactions between cytochrome *b*_5_ with P450s are known to enhance the coupling of the P450 ensemble and reduce the P450-dependent production of harmful reactive oxygen species [49,50,51]. Thus, the alcohol-induced decrease in cytochrome *b*_5_ levels could be implicated in the mechanisms underlying ethanol hepatotoxicity.

Considerable alcohol-induced changes in drug metabolism may also stem from alterations in the abundance and composition of UGTs. We observed significant upregulation of UGTs 1A6, 1A9, and 2A1; and significant downregulation of UGTs 1A3, 1A4, 2B7, 2B10, and 2B15 with alcohol exposure. Protein levels of UGT1A6 and UGT1A9, which are suggested to play roles in the non-oxidative metabolism of ethanol [52], were significantly elevated in HLMs from heavy drinkers compared to non-drinkers, a finding not previously reported. The effects of alcohol remained consistent even after excluding smokers from the analysis (Supplementary Table S2).

The expression patterns of important non-P450 and non-UGT enzymes and transporters involved in xenobiotic disposition were also altered by alcohol consumption. Whereas heavy alcohol intake induced the significant downregulation of AADAC, FMO3, FMO4, FMO5, and STS, the levels of FMO1 and MRP3 were significantly elevated. While STS is known for its role in metabolizing endogenous steroids rather than xenobiotics, alterations in STS expression are related to neurodegenerative disorders and hormone-sensitive cancers and holds clinical relevance as a drug target [53].

These findings somewhat contrast with those from a transcriptomics study of human liver samples from patients with alcohol-associated hepatitis, which concluded that drug-metabolizing enzymes were overall downregulated [54]. While our results do show a decrease in the majority of DMETs reported herein due to chronic alcohol exposure, we also measured notable increases in several DMETs. It is important to note that the results of proteomics analysis cannot be compared head-to-head with those of transcriptomics analysis. Transcriptomics analysis is known to have a higher level of background variability while quantitative proteomics is generally more reproducible, allowing for detection of biological effects at lower levels [32,41,55]. Additionally, protein expression often does not correlate with mRNA expression as it is regulated by a number of factors, as in the case of CYP2E1 which undergoes an alcohol-induced increase in protein stability [56].

In HLMs from moderate-to-heavy smokers, we observed a significant upregulation of CYP1A2, UGT1A6, and UGT2B4 alongside a significant downregulation of FMO3, FMO4, and FMO5 compared to non-smokers, independent of alcohol consumption. While CYP1A2 is known to be elevated with smoking, the literature on population-based studies into the potential induction of these enzymes in the liver due to smoking is limited.

Female donors exhibited significantly lower levels of CYP1A2, UGT2B7, UGT2B17, and UGT2B15, while displaying significantly higher levels of CYP2A6, UGT2A3, AADAC, FMO1, and STS compared to males. Although the association of UGT2B17 abundance with sex is well-known, the effect of sex on other clinically significant DMET proteins is a novel finding.

Limitations inherent to the study design permit only observational analysis and not a controlled environment. Consequently, confounding factors such as second-hand smoke could have contributed to elevated CYP1A1 or CYP1A2 levels in some individuals from the control group. However, the advantages of a population-based study include a realistic study design and the ability to detect significant changes despite inter-individual variations.

Previously, we provided evidence of an impact on CYP3A4, CYP1A2, and CYP2C19 activity due to the induction of CYP2E1 by alcohol [21,22], indicating the importance of consideration of the mutual functional effects of multiple enzymes constituting the P450 ensemble. The observed effects of alcohol on the induction or suppression of different P450s could have additional implications for the functioning of the drug-metabolizing ensemble as a whole. The use of the Provisional Index of Alcohol Exposure elaborated in this study for analyzing correlations between alcohol exposure and the function of drug-metabolizing enzymes in HLMs offers a promising avenue for the further exploration of the effects of alcohol on the drug-metabolizing system.

To our knowledge, this study represents the first comprehensive investigation of the effects of alcohol intake, smoking, and sex differences on the protein levels of important DMET proteins in a large set of human liver samples. Our data showed alcohol-associated differences in the abundance and composition of vital DMET proteins involved in the metabolism and disposition of various pharmaceuticals, highlighting the necessity of considering smoking tobacco and sex as covariates.

## Figures and Tables

**Table 1:** Characteristics of study population *.

	Females	Males
N = 94	33	61
Median Donor Age (Years)	62 (18–81)	68 (28–84)
Alcohol History		
Non-drinkers	13 (39%)	23 (38%)
Light Intake	2 (6%)	8 (13%)
Social Intake	12 (36%)	16 (26%)
Moderate Intake	5 (15%)	8 (13%)
Heavy Intake	1 (3%)	6 (10%)
Smoking Status		
Never Smokers	13 (39%)	15 (25%)
History of Smoking	12 (36%)	29 (47%)
Light smoking (<1 PPD)	2 (6%)	3 (5%)
Moderate Smoking (1–2 PPD)	5 (15%)	9 (15%)
Heavy Smoking (>2 PPD)	1 (3%)	5 (8%)
Race/Ethnicity		
White non-Hispanic	29 (88%)	59 (97%)
White Hispanic	3 (9%)	2 (3%)
African American	1 (3%)	0

* Values are expressed as the median and range or absolute numbers and percentages.

**Table 2:** TPA-based protein levels of major DMET proteins in HLMs of the study population.

*n*	Non-Drinkers		Heavy Drinkers		*p*-Value	Non-Smokers		Smokers > 1 ppd		*p*-Value	Males		Females		*p*-Value
	36		7			69		20			61		33		
	Mean ± SD		Mean ± SD			Mean ± SD		Mean ± SD			Mean ± SD		Mean ± SD		
*P450 protein levels (pmol/mg protein)*															
CYP1A1	1.0	±0.9	0.6	±0.1	0.013	0.7	±0.6	1.3	±1.7	0.10	0.9	±1.1	0.7	±0.6	0.43
CYP1A2	31.6	±13.2	13.5	±7.1	4.5 × 10^−5^	27.1	±13.0	39.3	±22.3	0.02	32.4	±17.4	24.0	±12.1	0.007
CYP2A6	67.8	±32.5	50.5	±26.1	0.15	66.8	±28.2	58.9	±30.5	0.28	59.5	±27.1	73.5	±29.4	0.03
CYP2B6	13.8	±9.4	32.7	±30.3	0.15	14.0	±8.5	18.6	±19.3	0.25	14.7	±13.9	14.6	±5.5	0.96
CYP2C8	25.5	±6.1	20.5	±13.9	0.39	24.6	±6.3	25.8	±9.0	0.77	24.2	±7.1	25.4	±6.5	0.44
CYP2C9	91.0	±25.0	60.9	±20.0	0.006	89.7	±23.1	85.5	±22.8	0.28	89.8	±22.9	84.1	±23.5	0.27
CYP2C18	4.8	±2.6	3.5	±2.5	0.18	4.7	±2.4	4.5	±1.8	0.55	4.6	±2.2	5.1	±2.3	0.27
CYP2C19	5.3	±3.8	4.2	±3.4	0.47	5.6	±3.8	6.2	±4.1	0.66	5.1	±3.4	6.8	±4.4	0.054
CYP2D6	28.8	±17.9	19.7	±11.2	0.10	27.3	±15.2	26.5	±19.7	0.79	25.7	±13.8	30.2	±19.5	0.24
CYP2E1	64.0	±17.2	108.0	±45.0	0.02	68.5	±25.3	63.6	±19.0	0.72	67.9	±25.5	65.7	±19.5	0.65
CYP3A4	67.0	±27.9	84.2	±62.2	0.25	68.5	±28.6	65.3	±45.6	0.79	62.8	±33.3	74.1	±30.8	0.10
CYP3A5	7.6	±14.3	10.0	±15.3	0.36	8.1	±13.4	9.6	±14.0	0.75	8.8	±13.1	9.0	±14.5	0.95
CYP3A7	0.9	±1.6	2.4	±4.3	0.21	0.9	±1.8	1.4	±2.1	0.21	1.1	±1.9	0.9	±1.5	0.57
CYP4A11	52.2	±13.7	31.4	±8.1	8.6 × 10^−5^	49.9	±14.5	47.1	±12.6	0.25	47.9	±13.5	51.0	±14.5	0.31
CYP4F2	33.7	±10.3	28.5	±10.3	0.25	32.7	±9.8	33.1	±9.0	0.97	32.4	±9.8	33.7	±8.6	0.49
*UGT protein levels (pmol/mg protein)*															
UGT1A1	28.2	±11.1	42.3	±19.4	0.054	27.1	±11.5	34.6	±17.4	0.08	28.7	±14.3	29.5	±10.9	0.76
UGT1A3	5.8	±3.7	4.4	±1.4	0.12	5.3	±3.9	5.9	±4.6	0.57	5.7	±4.4	4.6	±3.0	0.15
UGT1A4	74.1	±19.3	56.0	±14.1	0.014	69.3	±20.4	75.1	±20.4	0.32	71.0	±20.1	67.8	±21.4	0.48
UGT1A6	98.7	±24.5	147.4	±45.9	0.015	96.6	±29.0	117.7	±27.1	0.0013	106.5	±29.2	96.0	±38.1	0.17
UGT1A9	14.3	±3.8	19.6	±3.4	0.005	14.6	±4.4	15.7	±4.2	0.22	15.3	±4.3	15.5	±6.6	0.85
UGT2A1	1.7	±1.7	2.2	±1.8	0.51	1.2	±1.5	2.0	±1.7	0.054	1.4	±1.5	1.4	±1.6	0.80
UGT2A3	6.8	±3.0	8.2	±2.9	0.27	7.0	±3.1	6.9	±2.9	0.97	6.3	±2.7	8.6	±3.2	0.0010
UGT2B4	45.7	±10.5	45.0	±9.6	0.87	42.3	±9.0	49.8	±11.4	0.010	42.8	±9.1	46.1	±10.9	0.15
UGT2B7	205.2	±55.5	128.9	±31.8	0.0002	199.8	±54.2	195.6	±64.7	0.62	209.6	±56.2	177.7	±48.4	0.005
UGT2B10	15.8	±5.1	10.8	±4.9	0.037	15.7	±4.8	14.7	±7.0	0.47	15.3	±5.6	15.2	±5.3	0.95
UGT2B15	108.4	±30.6	75.9	±17.0	0.0006	107.4	±35.3	105.0	±43.3	0.67	118.7	±37.2	86.0	±21.6	5.7 × 10^−7^
UGT2B17	4.1	±5.8	4.9	±6.1	0.75	4.7	±5.6	5.2	±5.3	0.74	6.6	±6.0	1.8	±2.3	2.8 × 10^−7^
*non-P450, non-UGT enzymes (pmol/mg protein)*															
AADAC	31.4	±8.0	21.6	±3.8	7.3 × 10^−5^	30.3	±7.5	28.6	±7.9	0.30	27.9	±6.0	33.4	±8.9	0.003
CES1	344.0	±65.9	368.6	±96.4	0.54	335.7	±66.6	363.0	±74.4	0.16	338.8	±64.3	345.5	±74.9	0.67
CES2	33.6	±8.3	28.6	±11.2	0.30	35.6	±10.4	33.8	±11.6	0.40	35.2	±8.9	35.4	±12.9	0.93
CES3	0.4	±0.2	0.4	±0.1	0.60	0.4	±0.2	0.4	±0.1	0.71	0.4	±0.2	0.5	±0.1	0.12
FMO1	0.4	±0.1	0.5	±0.1	0.003	0.4	±0.1	0.4	±0.2	0.22	0.3	±0.1	0.4	±0.1	0.05
FMO3	69.9	±18.1	53.9	±13.0	0.02	71.9	±16.3	58.7	±21.0	0.011	69.1	±17.6	68.1	±18.4	0.82
FMO4	2.4	±0.6	1.9	±0.3	0.002	2.5	±0.6	2.1	±0.7	0.007	2.4	±0.6	2.4	±0.7	0.72
FMO5	47.6	±18.4	19.3	±3.2	1.0 × 10^−10^	45.3	±15.7	33.3	±12.1	0.0002	42.1	±16.6	42.5	±13.6	0.90
MGST1	341.6	±97.4	429.2	±146.9	0.17	312.6	±86.1	348.6	±124.5	0.15	329.9	±100.2	314.0	±97.4	0.46
MGST2	129.8	±27.8	137.3	±37.3	0.63	125.8	±26.3	130.6	±32.9	0.52	125.7	±28.2	132.4	±28.8	0.28
STS	0.7	±0.5	0.5	±0.1	0.006	0.7	±0.5	0.7	±0.4	0.56	0.6	±0.3	1.0	±0.7	0.002

Protein levels of metabolizing enzymes and drug transporters in hepatic microsomes from the 94 donors segregated by alcohol intake, smoking history, and sex (pmol/mg microsomal protein). Data provided as mean ± standard deviation (SD).

**Table 3:** Microsomal proteins exhibiting a significant correlation between their relative abundance and the PIAE *.

Protein ID	R	Student’s *t*-Test *p*-Value	Protein Name	FC ^a^	Cellular Location
HSPA5	0.656	7.2 × 10^−13^	Endoplasmic reticulum chaperone BiP	2.56	ER lumen, cytosol
FMO5	−0.600	1.7 × 10^−10^	Flavin-containing monooxygenase 5	0.40	ER membrane
POR	0.505	2.1 × 10^−7^	P450 reductase	1.52	ER membrane
HSPA9	0.491	5.1 × 10^−7^	Mortalin, Stress-70 protein	1.32	ER membrane
PDIA4	0.478	1.1 × 10^−6^	Protein disulfide-isomerase A4	1.44	ER lumen
VCP	0.454	4.2 × 10^−6^	Transitional endoplasmic reticulum ATPase	3.01	ER membrane
P4HB	0.443	7.9 × 10^−6^	Protein disulfide-isomerase	1.28	ER lumen
CYP4A11	−0.439	9.7 × 10^−6^	CYP4A11	0.57	ER membrane
HSP90B1	0.437	1.0 × 10^−5^	Endoplasmin, Heat shock protein 90	1.61	ER lumen
CYP2C9	−0.429	1.6 × 10^−5^	CYP2C9	0.61	ER membrane
GSTO1	0.426	1.8 × 10^−5^	Glutathione S-transferase omega-1	2.09	ER membrane
ERO1A	0.382	0.0002	ER oxidoreductase A	3.17	ER membrane
UGT1A4	−0.379	0.0002	UGT1A4	0.70	ER membrane
ERMP1	0.365	0.0003	Endoplasmic reticulum metallopeptidase 1	1.27	ER membrane
CYB5A	−0.360	0.0004	Cytochrome b_5_	0.82	ER membrane
ATP2A2	0.356	0.0004	Phospholipid-transporting ATPase IIA	1.34	ER membrane
UGT2B7	−0.343	0.0007	UGT2B7	0.56	ER membrane
UGT1A6	0.339	0.0008	UGT1A6	1.62	ER membrane
CYP2C8	−0.335	0.0009	CYP2C8	0.73	ER membrane
MGST1	0.335	0.0009	Microsomal glutathione S-transferase 1	1.39	ER membrane
FMO3	−0.333	0.0010	Flavin-containing monooxygenase 3	0.70	ER membrane
HERPUD1	0.323	0.0015	Homocysteine-responsive ER ubiquitin-like domain 1	1.66	ER membrane
CYP1A2	−0.318	0.0018	CYP1A2	0.41	ER membrane
MGST3	−0.317	0.0019	Microsomal glutathione S-transferase 3	0.74	ER membrane
UGT1A9	0.312	0.0022	UGT1A9	1.56	ER membrane
PDIA5	−0.309	0.0024	Protein disulfide-isomerase A5	0.71	ER lumen
AADAC	−0.309	0.0024	Arylacetamide deacetylase	0.75	ER membrane
ERAP2	0.307	0.0026	Endoplasmic reticulum aminopeptidase 2	1.57	ER membrane
CYP2J2	0.304	0.0028	CYP2J2	1.46	ER membrane
CYP2E1	0.302	0.0031	CYP2E1	1.52	ER membrane
CYP7B1	−0.293	0.0042	CYP7B1	0.81	ER membrane
PDIA6	0.286	0.0051	Protein disulfide-isomerase A6	1.15	ER lumen
HMOX1	0.282	0.0058	Heme oxygenase 1	1.49	ER membrane
UGT2B10	−0.279	0.0065	UGT2B10	0.57	ER membrane
UGT2A1	0.268	0.0090	UGT2A1	2.67	ER membrane
CYP4F11	−0.248	0.0159	CYP4F11	0.91	ER membrane
H6PD	0.241	0.0194	GDH/6PGL endoplasmic bifunctional protein	1.19	ER lumen
CYP2B6	0.232	0.0246	CYP2B6	1.72	ER membrane
UGT2B15	−0.227	0.0275	UGT2B15	0.66	ER membrane
UGT1A3	−0.226	0.0283	UGT1A3	0.51	ER membrane
CYP1A1	−0.223	0.0305	CYP1A1	0.00	ER membrane
ERLEC1	0.223	0.0309	Endoplasmic reticulum lectin 1	1.38	ER lumen

^a^ Arbitrary estimate of fractional change. Calculated as a ratio of averaged protein abundance for HLM samples with PIAE > 2.5 (*n* = 8) to that for the samples with PIAE < 1 (*n* = 35). * The proteins in the table are sorted in the order of increasing *p*-value. The positive values of the correlation coefficient are shown in red, while the green color designates the negative correlation. The proteins with *p*-value > 0.05 are not shown.

**Table 4:** Summary of the effects of alcohol exposure on the expression of drug-metabolized enzymes identified by PIAE analysis *.

Protein Class	Upregulated Proteins	Downregulated Proteins
Cytochrome P450	CYP2B6, CYP2E1, CYP2J2	CYP1A1, CYP1A2, CYP2C8, CYP2C9, CYP4A11, CYP4F11, CYP7B1
Cytochrome P450 partners	POR, HMOX1	CYB5A
UGTs	UGT1A6, UGT1A9, UGT2A1	UGT1A3, UGT1A4, UGT2B7, UGT2B10, UGT2B15
Other DMEs	GSTO1	AADAC, FMO3, FMO5, MGST1, MGST3

* The identifiers of the proteins revealed in the non-parametric analysis are underlined.

## Data Availability

The data are contained within the article. The raw datasets used to generate the reported results are available from the authors upon a reasonable request.

## Supplementary Materials

The following supporting information can be downloaded: https://www.mdpi.com/article/10.3390/jox15010020/s1, Figure S1: global and DMET proteome analysis of HLMs from light, social, and moderate alcohol drinkers; Figure S2: STRING analysis of significantly upregulated and downregulated proteins from global proteomics; Table S1: list of proteins localized to the endoplasmic reticulum used for normalizing DMET proteins; Table S2: TPA-based protein levels of major DMET proteins present in HLMs of heavy drinkers with >1 ppd smokers removed and smokers with heavy drinkers removed.
