# Supplementary material for: Effects of Chronic Alcohol Intake on the Composition of the Ensemble of Drug-Metabolizing Enzymes and Transporters in the Human Liver
Source: J Xenobiot. 2025 Jan 31;15(1):20. doi: 10.3390/jox15010020 (PMC11856820; doi:10.3390/jox15010020)
Supplement: Supplementary file 1 [file jox-15-00020-s001.zip › Supplementary Figures.pdf]

Effects of alcohol consumption and tobacco smoking on the composition of the ensemble of drug metabolizing enzymes and transporters in human liver

Kari A. Gaither, Dilip Kumar Singh, Guihua Yue, Julia Trudeau, Kannapiran Ponraj, Philip Lazarus, Dmitri R. Davydov, and Bhagwat Prasad

Supplementary figures

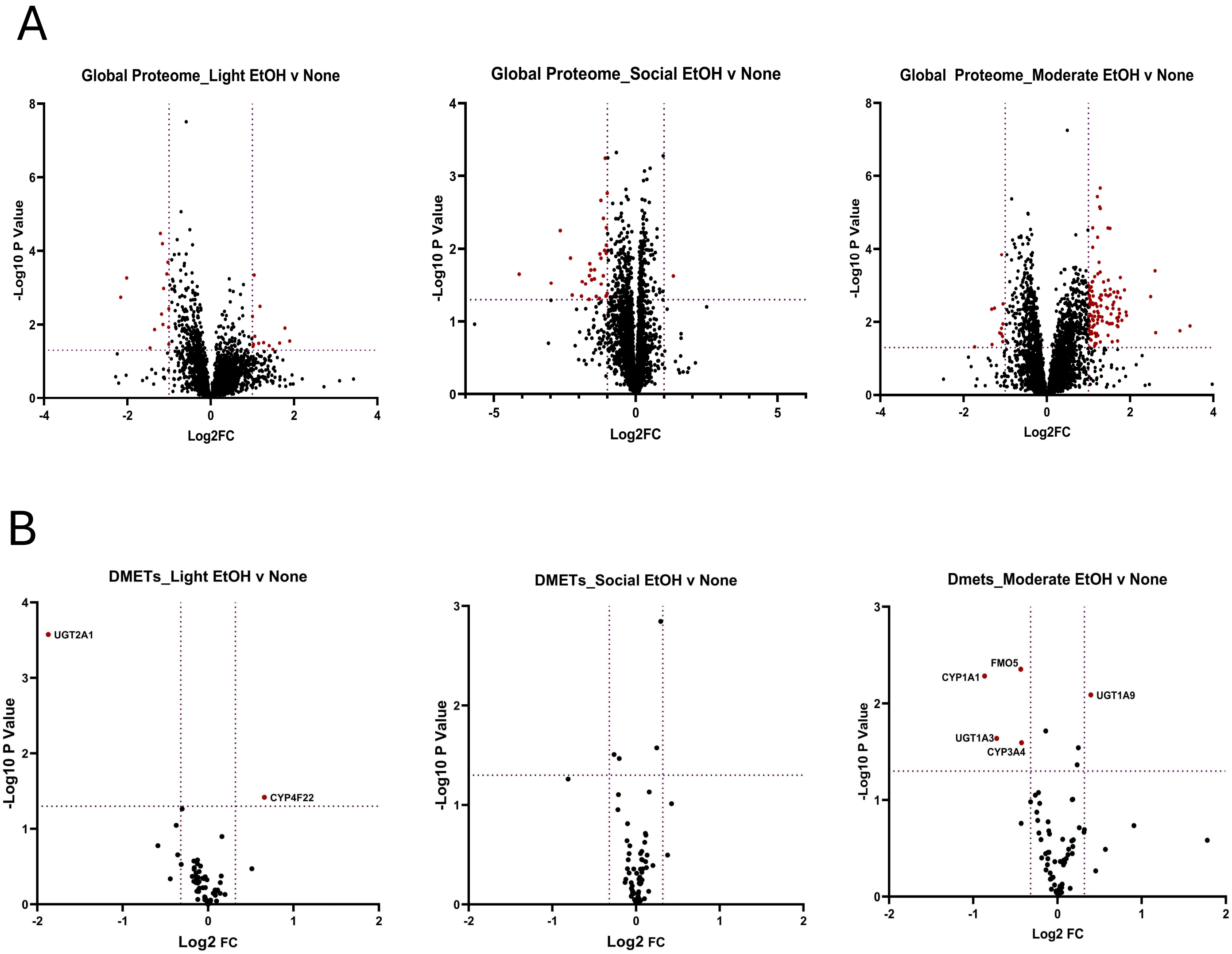

Figure S1. Global and DMET proteome analysis of HLM from light, social, and moderate alcohol drinkers. **(A)** Global proteomics of HLM from light drinkers vs non-drinkers showed little significant changes in proteins, similar to a comparison of social drinkers vs nondrinkers. There were moderate levels of significant differences in protein levels in HLM of moderate drinkers as compared to non-drinkers. (2.0 fold change (FC) cutoff, P-value<0.05) **(B)** The DMET proteome showed very little significant alteration in levels of expression in HLM from light drinkers, social drinkers, or moderate drinkers as compared to non-drinkers. (1.25 fold change cutoff, P-value<0.05)

A **KEGG Pathways Downregulated**

Description	Count in network	Strength	FDR
Taurine and hypotaurine metabolism	2 of 11	1.44	3.28E-02
Starch and sucrose metabolism	5 of 32	1.37	1.20E-04
Drug metabolism - cytochrome P450	7 of 64	1.22	1.63E-05
Cholesterol metabolism	5 of 48	1.19	5.10E-04
Oxidative phosphorylation	12 of 128	1.15	4.96E-08

B **KEGG Pathways Upregulated**

Description	Count in network	Strength	FDR
Proteasome	26 of 43	1.69	2.41E-30
Phenylalanine, tyrosine and tryptophan biosynthesis	3 of 6	1.61	1.20E-03
Valine, leucine and isoleucine degradation	22 of 46	1.59	6.63E-24
Propanoate metabolism	16 of 33	1.59	1.73E-17
Citrate cycle (TCA cycle)	12 of 28	1.54	8.50E-13

C

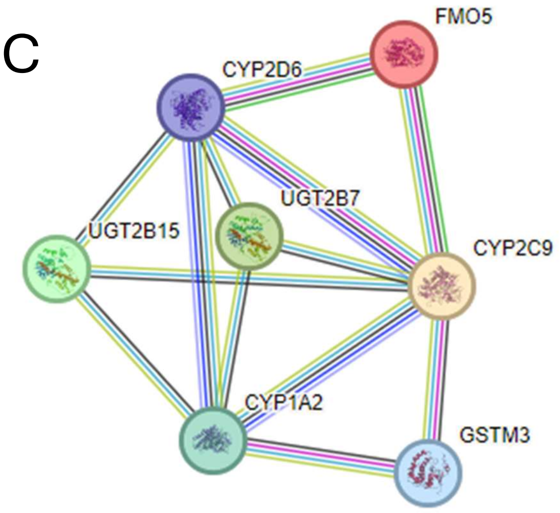

Figure S2. STRING analysis of significantly upregulated and downregulated proteins from global proteomics. **(A)** Downregulated KEGG Pathways include several key functions of the liver, including lipid metabolism, bile acid homeostasis, energy regulation and ATP synthesis, as well as drug metabolism. **(B)** Upregulated KEGG Pathways include those involved in proteolysis, amino acid metabolism, propanoate metabolism, and the TCA cycle. **(C)** Drug metabolism - cytochrome P450 KEGG Pathway. Seven members of this KEGG Pathway were significantly downregulated in the global proteomics data, indicating a strength of 1.22 and an FDR of 1.63×10^{-5}.
